# Supplementary material for: Quality of Life and Post-Surgical Complications in Patients on Chronic Antiplatelet Therapy with Proximal Femur Fracture: 12-Month Follow-Up after Implementing a Strategy to Shorten the Time to Surgery
Source: J Clin Med. 2023 Jan 31;12(3):1130. doi: 10.3390/jcm12031130 (PMC9918231; doi:10.3390/jcm12031130)
Supplement: Supplementary file 1 [file jcm-12-01130-s001.zip › jcm-2074559-supplementary.pdf]

Journal of Clinical Medicine

MDPI

Table S1. EQ-5D-5L index score and EQ-5D VAS assessment at different follow-up times.

Surgery	Pre-fracture			Preoperative			Ward			30 days			6 months			12 months			*p*
	Early *n* = 71	Delayed *n* = 57	Total *n* = 128	Early *n* = 71	Delayed *n* = 57	Total *n* = 128	Early *n* = 71	Delayed *n* = 57	Total *n* = 128	Early *n* = 55	Delayed *n* = 69	Total *n* = 124	Early *n* = 45	Delayed *n* = 60	Total *n* = 105	Early *n* = 42	Delayed *n* = 56	Total *n* = 98	
EQ-5D VAS																			
Mean (SD)	60.6 (2.7)	66.1 (2.4)	63.4 (1.8)	37.1 (2.4)	40.9 (2.1)	39.0 (1.6)	46.2 (2.5)	47.6 (2.2)	46.9 (1.7)	54.8 (2.6)	50.9 (2.2)	52.9 (1.7)	56.9 (3.0)	55.6 (2.6)	56.3 (1.9)	50.5 (3.8)	50.9 (3.3)	50.7 (2.5)	<0.001
EQ-5D-5L index																			
Mean (SD)	0.547 (0.044)	0.563 (0.038)	0.555 (0.029)	-0.378 (0.033)	-0.319 (0.028)	-0.349 (0.022)	0.057 (0.054)	0.174 (0.046)	0.115 (0.035)	0.355 (0.048)	0.333 (0.041)	0.344 (0.032)	0.418 (0.396)	0.396 (0.048)	0.407 (0.036)	0.325 (0.060)	0.356 (0.052)	0.340 (0.040)	<0.001
